# Hope in hardship: charting a new course for mental health in Afghanistan

**DOI:** 10.1192/bjo.2024.66

**Published:** 2024-05-07

**Authors:** Alvin Kuowei Tay

**Affiliations:** Discipline of Psychiatry and Mental Health, School of Clinical Medicine, University of New South Wales, Sydney, Australia

**Keywords:** Low- and middle-income countries, psychosocial interventions, mental health services, community mental health teams, human rights

## Abstract

The long legacy of upheavals and deprivations in Afghanistan and the associated mental health impacts on its people are well documented. A systematic review undertaken by Alemi et al (2023) presents the most comprehensive synthesis to date on this topic. Drawing on their findings, this editorial examines the complex mental health and psychosocial challenges confronted by neglected vulnerable groups such as pregnant and postnatal women, LGBTQ individuals, older adults, ethnic minority groups and Afghan refugees living overseas. It explores the potential challenges in rebuilding a resilient mental health system following the mass exodus of Afghanis. It calls for a whole-of-society approach that extends beyond clinical interventions to address the broader sociocultural and economic factors influencing mental health.

Afghanistan's history, marked by relentless conflict and political instability, has had a profound and lasting impact on the mental well-being of its people. The Soviet invasion in 1979, compounded by the rise of the Taliban regime and its stringent governance, reshaped the social fabric and contributed to widespread trauma and distress among the local population. The recent resurgence of the Taliban in 2021, following a brief period of relative stability, marked another significant turning point, undoing many of the advancements made in the preceding years.

These political upheavals were accompanied by the withdrawal of international forces and support, leading to an erosion of security and economic stability.^1^ The Taliban's takeover thrusted millions into conditions of extreme poverty and eroded social support systems that are vital for psychological resilience.^2^ The lack of necessities, coupled with the uncertainty and fear generated by ongoing conflict, the COVID-19 pandemic and exposure to natural calamities, have significantly increased the burden of mental health problems in the country.^3,4^

Furthermore, the protracted period of conflict has disrupted traditional community structures and family units, key elements in the Afghan culture that provide support and stability. The loss of loved ones to conflict, displacement from homes and the destruction of community networks have engendered lasting psychological impacts on individuals and communities. The collective trauma experienced over recent decades has been exacerbated by limited access to education, healthcare, social and mental health services, and basic rights for women and minorities.

## How can research shape mental health and psychosocial support?

This complex interplay of historical events, sociopolitical changes and economic challenges underscores the urgent need for comprehensive mental health and humanitarian assistance in Afghanistan. It highlights the necessity for interventions that are not only clinically effective but also culturally sensitive, acknowledging the unique historical context that shapes the mental health landscape of the Afghan people. Addressing these needs is essential for the nation's recovery and the well-being of its citizens, who have endured decades of upheaval and hardship.

The systematic review by Alemi et al^5^ in *BJPsych Open* makes a significant contribution to the literature on mental health in Afghanistan. This comprehensive analysis synthesises decades of research, providing a nuanced understanding of the complex mental health landscape in a country plagued by prolonged conflict and sociopolitical upheaval. The review highlights key areas such as the heightened vulnerability of specific groups (women, ethnic minorities, youth and people with disabilities), the impact of cultural factors on mental health, and the diverse range of coping mechanisms employed by Afghans. It also sheds light on the challenges faced by Afghan refugees and the implications of gender-based violence and substance use on mental health.

It utilised both peer-reviewed and grey literature, which enriches the breadth of perspectives and data included, and it concludes with actionable recommendations aimed at promoting health equity and sustainable care systems, which is a proactive approach to addressing the identified gaps.

The review's acknowledgement of not assessing the quality of studies and risk of bias is a critical limitation that could affect the validity of its conclusions. The need for deeper exploration into the impacts on specific vulnerable groups, such as children, women and refugees, is recognised. Additionally, although the review covers various interventions, it often lacks in-depth assessments of their long-term effectiveness and sustainability. Despite these limitations, the review significantly contributes to understanding the complex mental health needs within Afghan contexts, guiding future research and the development of culturally sensitive interventions and policies.

There is a clear necessity for future studies focusing on the quality and risk of bias in Afghan mental health research, particularly involving vulnerable groups and assessing long-term effectiveness of interventions. Such research should engage closely with all relevant stakeholders, including local academic institutions, non-governmental organisations (NGOs), Afghan leaders, minorities and vulnerable groups. This comprehensive approach, supported by international aid agencies and philanthropic organisations, is essential for developing targeted, effective and culturally sensitive mental health interventions.

In Afghanistan, the mental health crisis disproportionately affects various vulnerable groups. In addition to women, ethnic minorities, individuals with disabilities and youth, these groups extend to members of the LGBTQ community and older adults. These groups face heightened risks of mental health issues and suicide, exacerbated under the current Taliban regime. The challenges are compounded by pervasive stigma and fear of retaliation, making people less likely to seek help. LGBTQ individuals, in particular, grapple with severe societal and legal discrimination, leading to increased psychological distress and isolation. Older adults, often neglected in discussions of mental health, are also at significant risk, especially given the erosion of traditional family support structures due to conflict and displacement.

The expression of psychological distress in Afghanistan often aligns with cultural idioms, reflecting a deep reliance on faith and family. However, for these marginalised groups, the available coping mechanisms may be insufficient or inaccessible owing to societal exclusion and fear of stigma. This complex situation underscores the urgent need for inclusive and culturally sensitive mental health interventions that address the unique challenges faced by all vulnerable populations in Afghanistan.

## Child and maternal mental health

In Afghanistan, the mental health of children and adolescents is profoundly affected by communal and social peer violence, as well as restricted access to education. Research suggests that these factors, rather than war-related trauma, are primary contributors to mental health problems in young people. This scenario highlights the intricate relationship between clinical, social and educational elements in the development of trauma and stress-related disorders. The lack of educational opportunities not only hampers cognitive and social development but also exacerbates feelings of hopelessness and isolation among the youth, accentuating their vulnerability to mental health problems.^5^

Additionally, maternal mental health in Afghanistan is a critical concern, especially considering the extreme conditions of stress that many women face, as reflected in the high rates of postnatal depression.^6^ Access to adequate antenatal and postnatal mental healthcare is severely limited, which can have long-lasting effects on both mothers and their children. The mental well-being of mothers is crucial for the healthy psychological development of children, and the adverse impacts of maternal stress and trauma can be profound and enduring.

Furthermore, the country has seen a troubling rise in substance use, particularly heroin and methamphetamine, among its youth. This trend is closely linked with the prevailing mental health problems and is further complicated by the stigma associated with substance use, especially for women. Addressing these interconnected challenges requires a multifaceted and culturally sensitive approach to mental healthcare, one that encompasses the unique needs and circumstances of Afghan children, adolescents and mothers.

## Refugee mental health

The mass displacement of the population, a consequence of the prolonged conflict and recent political upheavals, raises serious mental health concerns among Afghan refugees. Many have been separated from their families and communities, facing the profound grief and anger that are associated with the loss of their homeland and the uncertainty of ever reuniting with their loved ones. This separation and loss are critical factors contributing to complex post-traumatic stress disorder among Afghan refugees.

The mental health challenges for these individuals are compounded by the uncertainties and difficulties of post-resettlement life. In their host countries, many Afghan refugees grapple with unstable residence status, loneliness and unemployment, all of which can intensify feelings of alienation and despair. Additionally, they often face discrimination and cultural barriers, making their adjustment even more challenging and affecting their mental well-being. For minority groups like the Hazaras, the situation is even more dire. With their culture at risk of extinction, the stress of displacement is accompanied by a deep sense of cultural loss and identity crisis. This results in complex post-traumatic symptoms, as they mourn not only personal losses but also the potential disappearance of their cultural heritage.^7^

These stressors, both pre- and post-resettlement, make recovery and adaptation a strenuous journey for Afghan refugees. Mental health support for this population needs to be multifaceted, acknowledging the complexity of their unique experiences. Comprehensive support mechanisms must address not only the clinical symptoms of mental disorders but also the broader sociocultural and existential challenges that these refugees face. This approach is vital for facilitating their recovery and integration into new communities, as emphasised by Alemi et al.^5^

## Building a community-based mental health and psychosocial support system

The recent mass exodus of Afghans, including many highly educated professionals following the Taliban takeover, has intensified the challenges faced by Afghanistan's healthcare system. This brain drain has left the country grappling with a critical shortage of skilled professionals, severely straining an already weak healthcare infrastructure. Efforts by the Afghan government and NGOs to bolster mental healthcare, through training medical practitioners and developing community-based psychosocial programmes, are commendable but are hindered by this acute shortage of qualified personnel.

The mental health system in Afghanistan, already struggling with limited access to services and inadequate staffing, faces additional hurdles due to the loss of these skilled professionals. The quality of care has consequently suffered and the capacity to implement well-designed interventions is significantly compromised. Community-based interventions, which focus on leveraging local cultural practices and social support systems, are crucial in this context. They offer a way to partially mitigate the impact of professional shortages by emphasising culturally sensitive approaches and community engagement in mental health.^5^

Implementing population-based epidemiological research using standardised instruments, such as the World Mental Health Survey Initiative, would yield invaluable insights into the mental health of the Afghan population. This approach would facilitate a longitudinal assessment of mental health trends among Afghan communities, aiding in the development of responsive interventions. Establishing such databases requires stringent adherence to privacy, ethical standards, governance and cultural sensitivity. Collaborative efforts with Afghan communities and leaders are vital to ensure the respectfulness and effectiveness of this initiative, making it a valuable resource for a global audience of researchers, policymakers and practitioners.

In conclusion, the mental health landscape in Afghanistan, shaped by its complex history and cultural diversity, requires a multipronged strategy. This strategy must include culturally attuned interventions, active community participation and comprehensive healthcare system reform. The integration of mental health into broader humanitarian and developmental initiatives is imperative, ensuring that mental health considerations are integral to Afghanistan's path towards recovery and resilience. This approach is crucial not only from a clinical perspective but also for addressing the broader sociocultural and economic factors influencing mental health. As Afghanistan confronts these myriad challenges, the need for both national and international cooperation is paramount to address the profound mental health crises and forge a future where every Afghan can achieve dignity and psychological well-being.

## Data Availability

Data availability is not applicable to this article as no new data were created or analysed in this study.
